# Evaluation of the inter and intraobserver reproducibility of the GRASP method: a goniometric method to measure the isolated glenohumeral range of motion in the shoulder joint

**DOI:** 10.1186/s40634-021-00352-z

**Published:** 2021-05-15

**Authors:** Miguel Angel Ruiz Ibán, Susana Alonso Güemes, Raquel Ruiz Díaz, Cristina Victoria Asenjo Gismero, Alejandro Lorente Gomez, Jorge Diaz Heredia

**Affiliations:** 1 Unidad de Hombro y Codo, Hospital Universitario Ramón y Cajal, Cta Colmenar km 9,100, 28046 Madrid, Spain; 2 Unidad de Hombro y Codo, Hospital FREMAP Majadahonda, Madrid, Spain

**Keywords:** Shoulder, Range of motion, Glenohumeral joint, Goniometric

## Abstract

**Purpose:**

To evaluate the intra and interobserver reproducibility of a new goniometric method for evaluating the isolated passive range of motion of the glenohumeral joint in an outpatient setting.

**Methods:**

This is a prospective observational study on healthy subjects. The Glenohumeral ROM Assessment with Scapular Pinch (GRASP) method is a new method for assessing the isolated range of motion (ROM) of the glenohumeral joint (GH) by a single examiner with a clinical goniometer. It measures the isolated glenohumeral passive abduction (GH-AB), passive external rotation (GH-ER) and internal rotation (GH-IR) with the arm at 45º of abduction.

These three GH ROM parameters were measured in both shoulders of 30 healthy volunteers (15 males/15 females, mean age:41.6[SD = 10.3] years). The full shoulder passive abduction, passive external rotation and internal rotation 45º of abduction were measured by the same examiners with a goniometer for comparison. One examiner made two evaluations and a second examiner made a third one. The primary outcome was the intra- and interobserver reproducibility of the measurements assessed with intraclass correlation coefficients (ICC) and the Bland–Altman plot.

**Results:**

The intra-observer ICC for isolated glenohumeral ROM were: 0.84 ± 0.07 for GH-ABD, 0.63 ± 0.09 for GH-ER, and 0.61 ± 0.14 for GH-IR. The inter-observer ICC for isolated glenohumeral ROM were: 0.86 ± 0.06 for GH-ABD, 0.68 ± 0.12 for GH-ER, and 0.62 ± 0.14 for GH-IR. These results were similar to those obtained for full shoulder ROM assessment with a goniometer.

**Conclusion:**

The GRASP method is reproducible for quick assessment of isolated glenohumeral ROM.

**Level of evidence:**

III

**Supplementary Information:**

The online version contains supplementary material available at 10.1186/s40634-021-00352-z.

## Background

The shoulder joint is a complex system in which four different joints (glenohumeral, acromioclavicular, sternoclavicular and scapulothoracic) work synchronically. All these different joints have a role in normal function, during sporting activities, and in shoulder pathology [18]. Certain problems affect more selectively one of these joints and the glenohumeral joint is affected predominately in different traumatic, overuse or degenerative shoulder problems [4, 11, 16, 20]. When there is a limitation of the shoulder range of motion (ROM), these joints can have different roles.

The importance of understanding the degree of involvement of the glenohumeral joint in the total shoulder ROM has been shown in the general population and in athletes for problems such as adhesive capsulitis[14, 16] or glenohumeral internal rotation deficit (GIRD) [19]. In both problems the full-shoulder ROM (FS-ROM) might be normal or only slightly changed as glenohumeral stiffness is masked by scapulothoracic hypermobility.

Some authors have suggested different methods for measuring the isolated passive glenohumeral ROM (GH-ROM) with a goniometer in clinic [6, 8]. These methods measure only passive external (PER) or internal rotation (PIR) and block scapular movements by pressing the coracoid and acromion to the bed (thus needing the subject to lie down) and require that the shoulder is placed in 90º of abduction. Lying completely or placing the shoulder in 90º of abduction is sometimes difficult to achieve in painful or older subjects. Although some clinicians assess glenohumeral ROM blocking the scapula with their hands, the so called “Codman´s grip”, this method has not been assessed properly and Nno reliable method has been developed to measure passive abduction (PABD) of the glenohumeral joint [24].

The main objective of this study was to evaluate the reproducibility in healthy adults of a new simple goniometric method, the Glenohumeral ROM Assessment with Scapular Pinch (GRASP), that measures selectively the PABD, PER and PIR of the glenohumeral joint and can be used by a single examiner using a simple clinical goniometer. The null hypothesis was that the presented method was not reproducible.

## Material and methods

### Measurement method

The Glenohumeral ROM Assessment with Scapular Pinch (GRASP) method was developed to allow for quick assessment of the passive abduction, external and internal rotation ROM of the glenohumeral joint with a goniometer by a single individual in a busy clinical setting.

**Fig. 1:**
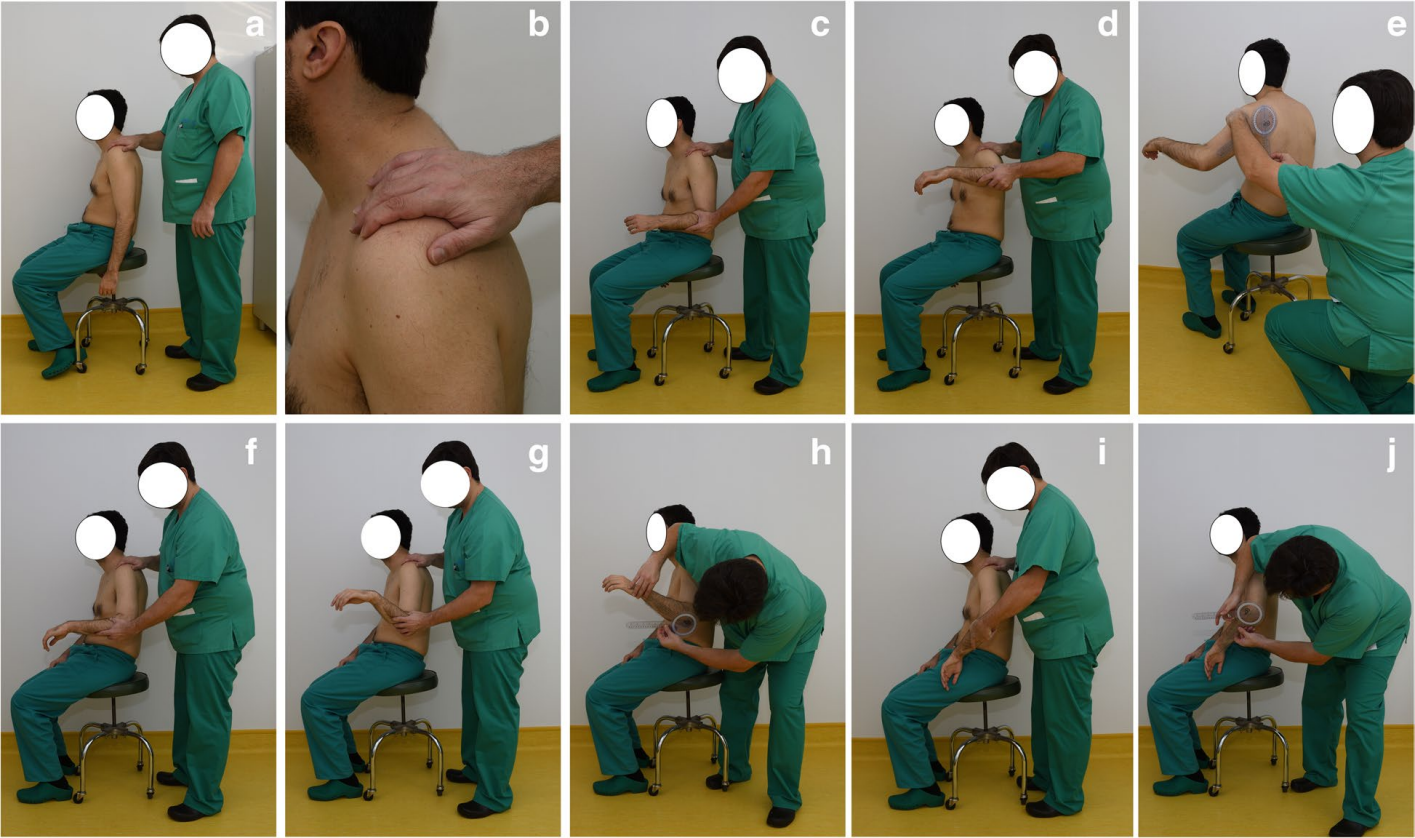
The Glenohumeral ROM Assessment with Scapular Pinch (GRASP) method. For a detailed description of each image see text


The subject to be measured is asked to undress from the thigh up and to sit in a stool (Fig. 1a). Then she/he is asked to relax and informed that no active movement is required from his/her side. For a left shoulder the examiner stands behind the subject, places his/her left hand over the shoulder, with the palm over the scapular spine, the thumb over the posterior aspect of the acromion and the rest of the fingers over the distal clavicle and the acromioclavicular joint (Fig. 1b). The osseous structures are felt delicately, and some soft but firm pressure is applied. The examiners picks up the elbow of the subject from posterior and flexes it to 90º so as to place it in 0º of rotation(Fig. 1c). Then passive abduction is started by the examiner using the right hand to elevate the elbow, taking care to block any scapular upward tilt with the left hand. Abduction is progressed until a firm stop is detected and scapular displacement starts (Fig. 1d). At that moment the examiner asks the subject to hold the arm in position, allowing for scapular movement if required but making sure abduction angle is maintained. Then the glenohumeral passive abduction (GH-PABD) angle is measured from the back with the goniometer, placing the goniometer in the coronal plane, with the vertex at the humeral head and one limb perpendicular to the floor and the other in line with the arm (Fig. 1e). Rotations are assessed in a similar manner: The scapula is blocked with the left hand and the arm is brought to 45º of glenohumeral abduction with the right hand on the elbow, keeping the 0º degrees of rotation, that is, parallel to the floor (Fig. 1f). Then the arm is externally rotated with the right hand while keeping notice of any scapular retraction. The movement is stopped when a firm stop is noticed and scapular displacement starts (Fig. 1g). At that moment the examiner asks the subject to hold the arm in position, then the glenohumeral passive external rotation (GH-PER) angle is measured from the lateral side in the plane of the forearm with the vertex of the goniometer placed at the elbow, one limb perpendicular to the coronal plne and the other in line with the elbow (Fig. 1h). The glenohumeral passive internal rotation (GH-PIR) angle is measured likewise but blocking scapular protraction (Fig. 1i and j). A measurement session can be seen in the video, Supplemental Digital Content 1.

**Additional file 1**: A video in mp4 format is included as SDC 1

### Sample size calculation

Based on the sample size formulas for estimating intraclass correlation coefficients with precision of Zou et al. [27] a minimum sample size of 58 was estimated for evaluating reproducibility. A total sample of 60 shoulder (30 subjects) was chosen.

### Subjects

Thirty healthy subjects (15 males and 15 females, mean age: 41.6 [standard deviation 10.3] years) with no history of shoulder pathology agreed to participate in this research study. Both shoulders´ GH-ROM and FS-ROM was measured in all subjects. Since agreement between measurements does not depend on the side in which measurements are made, right and left shoulder of the subjects were considered independently for a total of 60 shoulders.

Written informed consent to participate in the study was obtained for each subject. The study was approved by the Local Institutional Review Board (IRB approval number: 004/19).

### Data collection

Two examiners took part in data collection, both had extensive experience using the method. Three measurement sessions were completed for each 60 shoulders: two by examiner A (named sessions A1 and A2) and one by examiner B (named session B), following an A1–B–A2 sequence. The actual examiner assigned to the role of A and B was randomized for each patient with a flip of a coin. All sessions were planned in the same day. When one of the examiners was making the measurements, the other examiner left the room to be blinded to the results obtained by his/her fellow investigator.

Each measurement session included sequential measurements of the isolated glenohumeral passive abduction (GH-AB) ROM, passive external rotation (GH-ER) and internal rotation (GH-IR) with the arm at 45º of abduction, followed by measurement of the full passive shoulder ROM in abduction (FS-PABD), external rotation (FS-PER) and internal rotation (FS-PIR) with the arm at 45º of abduction in the sitting position. This second set of measurements was performed as a control for reproducibility. All measurements were performed with a set of long-arm (12″) goniometers (Physio Supplies Limited, Spalding, UK).

### Statistical analysis

The ROM values were tested for normality using the Kolmogorow-Smirnoff test. Intra and interobserver reproducibility was assessed for all six variables using Two Model intraclass correlation coeficients (ICC) [21]. ICC below 0.5 were considered poor, between 0.5 and 0.75 moderate, 0,75 to 0.9 good and > 0.9 excellent [12]. For intraoberver reproducibility the data from sessions A1 and A2 were compared. For interoberver reproducibility the data from sessions A1 and A2 were compared with B. Bland-Altmann plots were obtained for all six variables [5].

An arbitrary cut-off point of 10º was stablished to further assess reproducibility: the percentage of measures that varied more than 10º between repeated measurements was calculated. The minimal clinically important difference (MCID) in ROM for shoulder movements has not been clearly established, but a range of 2º to 10º has been proposed [10, 22].

## Results

The mean [standard deviation] values of the six angular parameters measured were: FS-ABD.176º[6.57º]; FS-ER:79.6º[8.94º]; FS-IR:77.8º[7.59º]; GH-ABD: 81.4º[8.94º]; GH-ER: 61.0º[11.8º]: and GH-IR: 49.9º[10.7º].

**Fig. 2:**
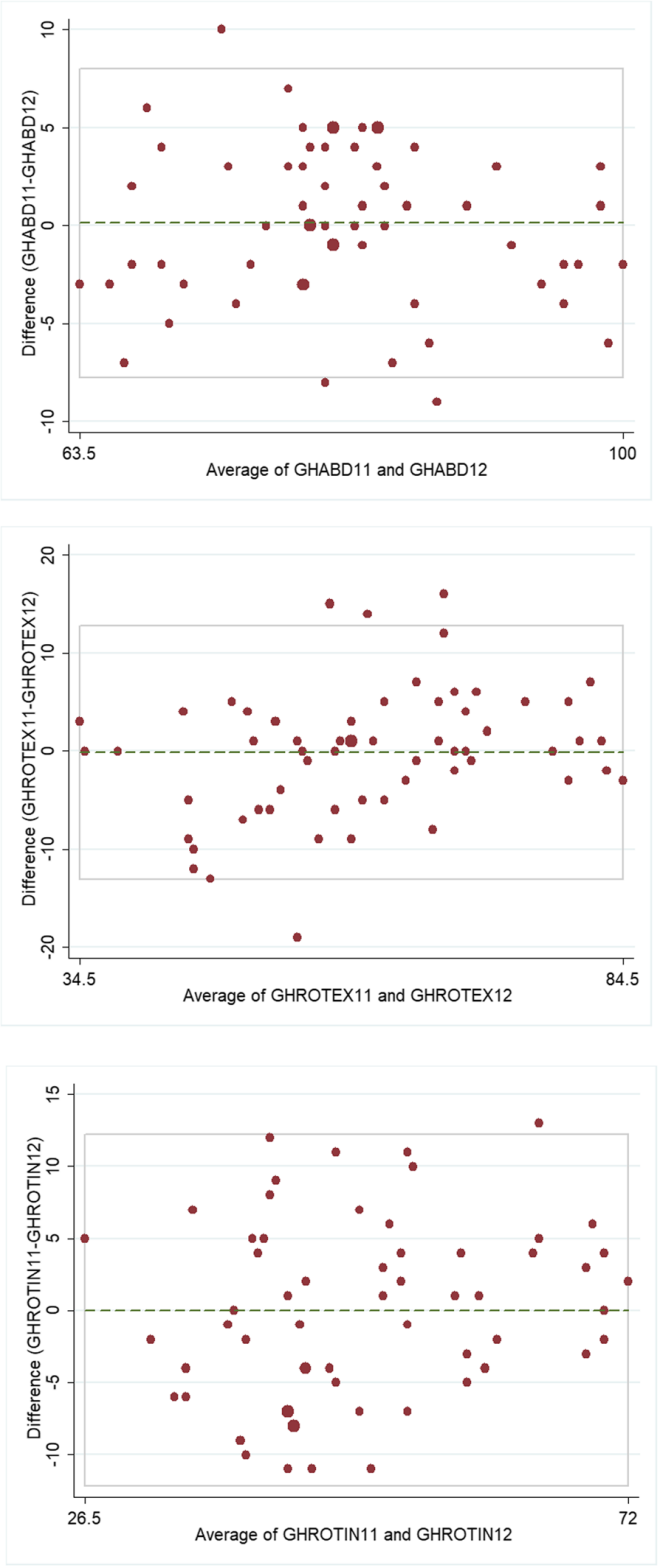
The Bland–Altman plots for intraobserver (**a**, **b**, **c**) and interobserver (**d**, **e**, **f**) reproducibility for glenohumeral passive abduction (GH-PAS), external rotation (GH-PER) and internal rotation (GH-PIR)

The ICC obtained for intra and interobserver reproducibility can be seen in Table 1. There was good agreement in the abduction measurements. For the rotation measurements the agreement was moderate. The results obtained for GH measurements were at least as good as those obtained for FS measurements for all three measured angles. The Bland–Altman plots for all three GH angles measured can be seen in Fig. 2. The percentage of measurements with variations below 10º between examiners was 97% for GH-PAB and 85% for both GH-PER and GH-PIR.

**Table 1:** Intra and interobserver Intraclass Correlation Coefficients (ICC) with 95% confidence intervals (95% Conf. Int) for the measurements of isolated glenohumeral passive abduction (GH-AB) ROM, passive external rotation GH-(ER) and internal rotation (GH-IR) with the arm at 45º of abduction; and for full shoulder (FS) passive abduction (FS-AB) ROM, passive external rotation (FS-ER) and passive internal rotation (FS-IR) with the arm at 45º of abduction are also presented. ICC values below 0.5 (considered poor reproducibility) are shaded in dark grey, values between 0.5 and 0.75 (moderate reproducibility) are shaded in light grey,and values over 0,75 (good reproducibility) are left white

	Intraobserver		Interobserver	
Variable	ICC	95% Conf. Int	ICC	95% Conf. Int
GH-PAB	0.84	0.75–0.91	0.86	0.77–0.92
GH-PER	0.63	0.52–0.80	0.68	0.52–0.80
GH_PIR	0.61	0.44–0.75	0.62	0.45–0.76
FS-PAB	0.81	0.70–0.89	0.83	0.72–0.90
FS-PER	0.80	0.68–0,88	0.63	0.47–0,77
FS-PIR	0.57	0.41–0.73	0.53	0.36–0.70

## Discussion

The most important finding of this study is that the Glenohumeral ROM Assessment with Scapular Pinch (GRASP) method for the measurement of isolated passive glenohumeral ROM has reproducibility in line with other standard techniques used in clinical practice to assess full-shoulder passive range of motion. It provides very good reproducibility in abduction measurements and good acceptable reproducibility in external and internal rotation.

A precise assessment of the role that each individual joint has in the full shoulder ROM is something that is rarely done in the typical busy clinical setting [17]. The main exception is glenohumeral internal rotation in subjects with GIRD, a problem that affects overhead athletes such as swimmers [3, 7, 23] and tennis [7, 8, 23] players; in these subjects an imbalance between glenohumeral internal and external rotation causes symptoms and a precise assessment of isolated GH-ROM is needed. In other problems, such as adhesive capsulitis in which the glenohumeral joint is selectively affected, a precise measurement of the isolated GH-ROM could be useful, as it is well-recognized that subjects with adhesive capsulitis use their scapulothoracic joint to compensate for GH-ROM loss, making precise assessment of the clinical course of the disease difficult, as an improvement in FS-ROM could be attributed to progressive healing of the capsular problem but it might also be due to increased scapulothoracic compensation. Thus, a simple method to assess GH-ROM should be of help.

Unfortunately, the available tools to assess reliably the GH-ROM have significant limitations that make them unusable in a typical clinical setting. Systems that use cumbersome motion tracking systems [1, 2], fluoroscopy [9], scapular immobilizers [15] or even percutaneous bone pins [13] have been used in experimental settings and have shown excellent reproducibility. Other authors have presented systems in which an examiner manually immobilises the scapula in supine [23, 25] position while another examiner measures GH-ER and GH-IR. These systems are widely used [19] but have very poor reproducibility, with ICC values below 0.5–0.6 [6, 26]; furthermore they require that the arm is placed in 90º of abduction, a position that might be painful for many subjects with shoulder problems. Some clinicians assess glenohumeral ROM manually blocking the scapula with their hands, the so called “Codman´s grip”, this method has not been properly validated and the GRASP method built on this clinical exam method trying to standardize it,

The new method presented here was developed with the following requirement in mind: it should use only a manual goniometer (available in virtually any outpatient clinic), it should require only one examiner, it should be quick to use and painless to the patient and should be as reproducible as the usual methods to assess ROM in a busy clinical setting. The GRASP method has clear advantages for the clinician: it is simple to use, requires only one examiner, uses a simple goniometer, does not require the patient to lie or place the shoulder in 90º of abduction (some healthy subjects do not reach 90º of isolated passive glenohumeral abduction and many injured shoulders are very uncomfortable at thisextreme ROM), the measurements are easy to interpret and comparisons to the contralateral side or previous exams can be done seamlessly.

The intra and interobserver reproducibility of the GRASP method to assess GH-ROM described are in par with the measurements we took of FS-ROM. This suggest that it might be as adequate as the typical methods that we use in our practice to assess ROM. The ICC values for GH-ABD are especially relevant, with 95% confidence intervals over 0.75, the reproducibility should be considered good and there is no other system, to our knowledge, that allows for GH passive abduction assessment in the clinical setting. The values for GH-ER (0.68) and for GH-IR (0.62) are also acceptable but lower than for abduction, but precise assessment of rotation movements is more difficult [24]. The method presented has similar ICC ranges as the FS-ER and FS-IR measurements presented here and in line, if not better, than those presented by other authors that assess isolated GH rotational ROM: assessments: Boone and Smith [6] tested a supine, manual goniometer, two examiner, manual scapular stabilization method in 50 healthy athletes and found intraobserver ICCs of 0.58 for ER and 0.60 for IR, and interobserver ICCs of 0.78 for ER and 0.38 for IR. Wilk et al. [26] evaluated the reproducibility of three different ways to stabilize the scapula in the supine position during GH-IR measurement and found interobserver ICC consistently below 0.5. thus, this new system seems to be clearly more reproducible than previous methods.

The ICC values obtained are not optimal, this being a limitation of this method, as this tool might be of limited use when very precise assessment is needed. Despite of this, using the MCID threshold of 10º showed that abduction and probably external rotation can be measured relatively safely with this new method and it seems as reproducible if not more reproducible as other available methods. A clear limitation of the measurement method is that it requires the subject to keep the shoulder in 45º of active adduction during rotational measurements, some patients, with significant rotator cuff problems might struggle to keep the arm in that position for long. Another limitation of this study is sample size: although a sample size calculation for the reproducibility analysis was indeed performed, the results obtained are limited in scope regarding the adquisition of normative data for healthy individuals, as the sample size is too small and includes a relatively young population. Ideally further reproducibility assessment could have been performed making repeated measurements in different days, this was not done and is a limitation of this study. To finish, a clinical tool should not be only accurate, but it should also be useful; this study only evaluates reproducibility, it lacks a clear clinical indication for use; this is a limitation that our research team is working to solve.

## Conclusions

The Glenohumeral ROM Assessment with Scapular Pinch (GRASP) method is an easy to use, reproducible method for quick and assessment of the isolated glenohumeral range of motion in healthy adults. The quality of the data obtained is similar to that obtained when assessing full shoulder passive ROM with a goniometer.

## Data Availability

The datasets generated and/or analysed during the current study are not publicly available but are available from the corresponding author on reasonable request.
